# Lateral Flow Immunochromatographic Assay for Detection of Antibodies Against a Recombinant Human Adenovirus Type 3 Hexon Fragment Using Gold Nanoparticles and Quantum Dots

**DOI:** 10.3390/diagnostics16162670

**Published:** 2026-08-21

**Authors:** Kanatbek Mukantayev, Kanat Tursunov, Laura Tokhtarova, Bisultan Abirbekov

**Affiliations:** Laboratory of Immunochemistry and Immunobiotechnology, National Center of Biotechnology, 13/5, Kurgalzhynskoye Road, Astana 010000, Kazakhstan; mukantaev@biocenter.kz (K.M.); laura_2626@mail.ru (L.T.); abirbekovbisultan@gmail.com (B.A.)

**Keywords:** human adenovirus, lateral flow immunochromatographic assay, hexon protein, gold nanoparticles, quantum dots

## Abstract

**Background/Objectives**: Human adenoviruses (HAdVs) are widely used as vectors for vaccines and gene therapy; however, pre-existing immunity can reduce their efficacy. Therefore, rapid and accessible serological methods are required to assess antibody levels against adenoviruses. Lateral flow immunochromatographic assay (LFIA) sensitivity depends on the label and antigen. In this study, we aimed to develop and evaluate LFIA systems based on gold nanoparticles (GNPs) and quantum dots (QDs) using a recombinant HAdV hexon protein. **Methods**: A recombinant HAdV hexon protein fragment (rhHAdV, 35 kDa; amino acids A120-R316) was expressed in *Escherichia coli* and purified using Ni^2+^ affinity chromatography. Protein identity was confirmed using sodium dodecyl sulfate–polyacrylamide gel electrophoresis, Western blotting, and liquid chromatography–tandem mass spectrometry analyses. Two LFIA formats were developed using Protein G-conjugated GNPs (GNP-G) and QDs (QD-G). Agreement with the reference ELISA was evaluated using 90 human serum samples. **Results**: The rhHAdV antigen demonstrated reactivity with anti-adenovirus-positive human sera in ELISA. Antibody detection was achieved at serum dilutions of up to 1:300 and 1:1000 for the GNP- and QD-based LFIA, respectively. The GNP-G and QD-G LFIA formats demonstrated positive percent agreements of 96.9% and 100%, respectively, while both assays showed a negative percent agreement of 98.2% compared with the reference ELISA. **Conclusions**: The findings demonstrate the feasibility of a QD-based LFIA for qualitative detection of antibodies reactive with the recombinant HAdV-B3 hexon fragment.

## 1. Introduction

Human adenoviruses (HAdVs) comprise multiple species and serotypes that are associated with a wide range of respiratory, ocular, and gastrointestinal diseases. Owing to their immunogenicity and structural characteristics, adenoviruses have also been extensively studied as vaccine vectors and gene delivery systems. In both clinical diagnostics and epidemiological investigations, serological methods are valuable for assessing previous exposure to adenoviruses and population seroprevalence [1]. Adenoviral vectors have been widely used as delivery systems for vaccine development and gene therapy because of their favorable structural and functional characteristics [2,3]. Accordingly, reliable serological assays are important for epidemiological studies, seroprevalence surveys, and evaluation of previous adenovirus exposure.

In modern laboratory practice, adenovirus infection and previous immune exposure are commonly evaluated using polymerase chain reaction (PCR) and enzyme-linked immunosorbent assay (ELISA), depending on the clinical objective. Alternatively, the lateral flow immunochromatographic assay (LFIA) represents a single-step diagnostic approach that does not require complex equipment, additional reagent preparation, or prolonged sample processing [4]. The structural and technological features of the LFIA ensure rapid testing and straightforward interpretation of results, which are particularly advantageous when screening large populations at the point of care or in field settings [5]. In addition, LFIAs are characterized by cost-effectiveness, operational simplicity, and suitability for point-of-care testing [6]. Furthermore, LFIAs are widely applied in the diagnosis of bacterial, viral, and parasitic infections [4,5,6,7,8]. Overall, LFIA represents a promising platform for rapid serological testing because of its simplicity, short turnaround time, and suitability for epidemiological investigations and decentralized testing. Recent research has focused on improving the sensitivity of the LFIA and developing quantitative assay formats. For instance, studies have evaluated the use of novel labels, such as quantum dots (QDs) [9], which exhibit high stability, a large extinction coefficient, high quantum yield, and prolonged fluorescence lifetime. Accordingly, QDs are excellent labels that can be conjugated with antibodies to develop highly sensitive LFIA-based diagnostic systems [10,11].

While many LFIA platforms are designed for the direct detection of pathogens or antigens, serological immunochromatographic assays provide an opportunity to detect antibodies generated following previous adenovirus exposure. Such assays may support seroepidemiological studies and retrospective assessment of exposure to specific adenovirus antigens. In the present study, a recombinant fragment of the human adenovirus type 3 (rhHAdV) hexon protein (amino acid residues A120-R316) was selected as the target antigen for antibody detection. This fragment was selected based on previous studies indicating that it contains immunoreactive domains suitable for serological applications while remaining amenable to recombinant expression in *Escherichia coli*. Its selection was guided by published information on the antigenic organization of the adenovirus hexon protein rather than by structural modeling or epitope mapping performed in the present study.

We hypothesized that an LFIA based on rhHAdV would enable reliable detection of binding antibodies directed against the recombinant HAdV-B3 hexon fragment and that the use of quantum dots as signal-generating labels would provide greater dilutional detectability compared with conventional gold nanoparticles (GNPs). To our knowledge, this is one of the first studies to directly compare GNP- and QD-based LFIA formats employing the same rhHAdV and identical clinical serum samples for anti-adenovirus IgG detection. Therefore, the aim of the present study was to develop and evaluate GNP- and QD-based LFIA formats employing a rhHAdV for the rapid serological detection of anti-adenovirus IgG antibodies.

## 2. Materials and Methods

### 2.1. Bacterial Strains and Plasmids

*Escherichia coli* strains BL21 (Thermo Fisher Scientific, Carlsbad, CA, USA, ECO114) and DH5α (Thermo Fisher Scientific, Carlsbad, CA, USA, ECO112), as well as the pET28 plasmid (Novagen, Merck, Darmstadt, Germany, 69864-3), were used in the study. Recombinant proteins were purified using Ni^2+^-NTA affinity chromatography columns (Cytiva, Uppsala, Sweden, 17524701). For Western blot analyses, mouse monoclonal antibodies against the His-tag (Thermo Fisher Scientific, Waltham, MA, USA) (RRID: AB_557403) and species-specific secondary antibodies conjugated with horseradish peroxidase (Sigma-Aldrich, St. Louis, MO, USA) were used. Human anti-adenovirus IgG antibodies were detected using a commercial human adenovirus IgG (ADV IgG) ELISA Kit (Abbexa Ltd., Cambridge, UK; Catalog No. abx364854). The assay is a qualitative indirect ELISA intended for the detection of human anti-adenovirus IgG antibodies in serum samples. All assays were performed according to the manufacturer’s instructions. Samples were classified as positive or negative according to the manufacturer’s cut-off criteria. No equivocal results were obtained; therefore, all samples were assigned to either the positive or negative group for comparative analysis.

### 2.2. Construction of the Recombinant HAdV-B3 Hexon Plasmid

The recombinant HAdV-B3 hexon plasmid encoded amino acid residues A120-R316 of the human adenovirus type 3 hexon protein (GenBank accession BAF42828.1). The DNA fragment encoding this region was codon-optimized for expression in *E. coli*, chemically synthesized, and cloned into the pET-28a(+) expression vector between the NcoI and XhoI restriction sites to generate an N-terminal His-tag fusion protein. The integrity of the recombinant construct was confirmed by Sanger sequencing prior to transformation into *E. coli* BL21(DE3). The amino acid sequence of the recombinant HAdV-B3 hexon fragment is provided in Supplementary Tables S1 and S2.

### 2.3. Expression of rhHAdV

Transformed *E. coli* BL21 cells were cultured in 5 mL of Luria–Bertani (LB) broth supplemented with kanamycin (Sigma-Aldrich, St. Louis, MO, USA; Cat. No. K4000) at 37 °C with shaking at 160 rpm. Upon reaching the logarithmic growth phase, isopropyl-β-d-1-thiogalactopyranoside (IPTG) (Sigma-Aldrich, Cat. No I6758-1G) was added to the culture at final concentrations of 0.2, 0.5, 1, and 5 mM to induce protein expression. The induced cells were further incubated at 37 °C with shaking at 160 rpm. Protein expression was induced when the culture reached an optical density at 600 nm (OD600) of approximately 0.6–0.8.

Aliquots (100 µL) of the *E. coli* culture were collected every 2 h post-induction. Cells were harvested by centrifugation at 10,000× *g* for 5 min at 4 °C. The resulting pellets were resuspended in 1 mL of buffer (20 mM HEPES, pH 7.5; 150 mM NaCl) and lysed using an OMNI Ruptor 4000 ultrasonic homogenizer (OMNI International, Kennesaw, GA, USA). Sonication was performed twice for 30 s at maximum power. The lysates were subsequently analyzed using sodium dodecyl sulfate–polyacrylamide gel electrophoresis (SDS-PAGE).

The producer strain was cultured in 5 mL of LB broth supplemented with the appropriate antibiotic for 12 h at 37 °C with constant agitation. Subsequently, 50 µL of the overnight culture was inoculated into 50 mL of LB broth containing the antibiotic and incubated at 37 °C until the logarithmic growth phase was reached. Upon attaining the desired cell density, IPTG was added to a final concentration of 0.2 mM to induce protein expression. The induced cells were further cultured for 16 h at 37 °C. Protein expression was induced when the culture reached an optical density at 600 nm (OD600) of approximately 0.6–0.8. Cell biomass was harvested by centrifuging 50 mL of the culture at 3000× *g* for 20 min. The cell pellet was resuspended in one-third of the volume of physiological saline and centrifuged again under the same conditions. The resulting pellet was then resuspended in 10 mL of TNE buffer (20 mM Tris, pH 7.5; 1 mM EDTA; 100 mM NaCl). The cells were disrupted by sonication (22 kHz, 4 cycles of 120 s each) and centrifuged at 17,000× *g* for 60 min. The pellet was resuspended in 10 mL of buffer 1 (20 mM HEPES, 500 mM NaCl, 1 M urea) and incubated for 30 min on an orbital shaker at 25 °C, followed by centrifugation at 17,000× *g* for 30 min. The resulting pellet was resuspended in metal-affinity chromatography binding buffer (20 mM HEPES, 500 mM NaCl, 20 mM imidazole, 8 M urea, 10 mM 2-mercaptoethanol) and subjected to sonication (22 kHz, 60 s). After sonication, the suspension was incubated for 30 min on an orbital shaker at 25 °C and then centrifuged at 3000× *g* for 20 min.

The selected expression conditions were used for large-scale production of the recombinant hexon protein. Following induction and cultivation, the recombinant protein was purified from the bacterial lysate, subjected to refolding, and subsequently characterized using biochemical and proteomic approaches. The recombinant protein was purified from the supernatant using a Ni^2+^-NTA affinity column (Cytiva, Uppsala, Sweden, 17524701). Elution was performed using the same buffer containing 8 M urea with a linear imidazole gradient (20–500 mM). The final yield of purified rhHAdV after purification and refolding was approximately 500 μg/mL. Protein refolding was carried out by dialysis against a 300-fold excess volume of 25 mM sodium phosphate buffer (pH 7.25) containing 300 mM NaCl. The refolded protein was analyzed by denaturing electrophoresis in 15% SDS-PAGE gel. Western blot and LC-MS/MS were employed to evaluate the purified recombinant proteins.

### 2.4. Conjugation of GNPs with Protein G

Commercial colloidal GNPs with an average diameter of 20 nm (Sigma-Aldrich, Darmstadt, Germany) were used in this study. The nanoparticles were supplied as a standardized preparation and used according to the manufacturer’s recommendations without additional physicochemical characterization. The obtained GNP solution was cooled and stored at 4 °C. Prior to conjugation, the pH of the GNP solution was adjusted to 8.5–9.0 using potassium carbonate. Protein G (Thermo Fisher Scientific, Waltham, MA, USA; Cat. No. 21193) was added to the pH-adjusted GNP suspension to prepare the GNP–Protein G conjugate used throughout this study. The mixture was incubated for 60 min at 25 °C, after which 250 µL of 10% bovine serum albumin (BSA) was added. The suspension was further incubated for 10 min at 25 °C and subsequently centrifuged at 8000× *g* for 30 min. The resulting pellet was resuspended in 1.5 mL of phosphate-buffered saline (PBS) containing 0.25% BSA.

### 2.5. Conjugation of QDs with Protein G

Carboxyl-functionalized Qdot™ 585 nanocrystals (Invitrogen, Carlsbad, CA, USA), with an emission maximum at 585 nm and a broad excitation spectrum extending into the ultraviolet region, were used for conjugation with Protein G according to the previously described protocol [12], with minor modifications. Briefly, 60 µL of the QD suspension was added to 840 µL of 0.05 M 2-(*N*-morpholino)ethanesulfonic acid (MES) buffer (pH 5.8; Tocris Bioscience, Bristol, United Kingdom, Cat. No. 5887). To activate the carboxyl groups on the surface of the QDs, 50 µL of *N*-(3-dimethylaminopropyl)-*N*′-ethylcarbodiimide hydrochloride (EDC, HiMedia, Mumbai, India, RM10170) and 50 µL of *N*-hydroxysulfosuccinimide sodium salt (NHS) (Glentham Life Sciences, Corsham SN13 9SW, United Kingdom, GE3858), both at 10 mg/mL, were added to the mixture. The resulting suspension was incubated for 30 min at 25 °C. Subsequently, 400 µL of Protein G solution (1 mg/mL in PBS, pH 7.2) was added to the activated QDs, providing a molar ratio of 8:1. The reaction mixture was incubated for 2 h under continuous agitation using a PSU-10i orbital shaker (Biosan, Riga, Latvia) under light-protected conditions to prevent QD photodegradation and ensure efficient conjugation. Following incubation, the conjugate was purified to remove excess activating reagents and unbound Protein G. Purification was performed by concentration and dialysis against 10 mM borate buffer (pH 8.6) using Amicon Ultracel 30 K centrifugal filters (Millipore, Burlington, MA, USA) at 10,000× *g* for 15 min. After purification, the conjugate was centrifuged at 15,000× *g* for 20 min at 4 °C. The resulting pellet was resuspended in 400 µL of stabilizing buffer (20 mM Tris-HCl, pH 8.0; 0.5% BSA; 0.1% Tween 20; 5% sucrose; 0.05% NaN_3_). The purified and concentrated conjugate was stored at 4 °C until further use.

### 2.6. Assembly of LFIA

GNP–Protein G (GNP-G) and QD–Protein G (QD-G) conjugates were applied onto separate PT-R5 membranes (Advanced Microdevices Pvt. Ltd., Haryana, India) at a volume of 11 µL per 1 cm strip using an AirJet Quanti 3000 dispensing system (XYZ3050 platform, BioDot, Irvine, CA, USA). Test and control lines were dispensed onto a laminated nitrocellulose membrane (CNPC-SS12-L2-H50, Advanced Microdevices Pvt. Ltd., Haryana, India) using a FrontLine 1000 dispensing system (XYZ3050 platform, BioDot). The test line consisted of purified rhHAdV immobilized at a working concentration of 1 mg/mL in PBS containing 10% glycerol. This concentration was selected during preliminary assay development and was used throughout all subsequent experiments. The control line consisted of monoclonal mouse anti-human IgG (Fc-specific) antibodies (clone HP6017; Sigma-Aldrich, Cat. No. MAB1302) immobilized at a concentration of 1 mg/mL in PBS containing 10% glycerol. During chromatographic migration, excess Protein G-conjugated nanoparticles bound to human IgG molecules are captured by the immobilized anti-human IgG antibodies through recognition of the Fc region of human IgG, thereby generating the control line and confirming proper sample migration and assay functionality independently of antigen-specific binding at the test line.

**Figure 1:**
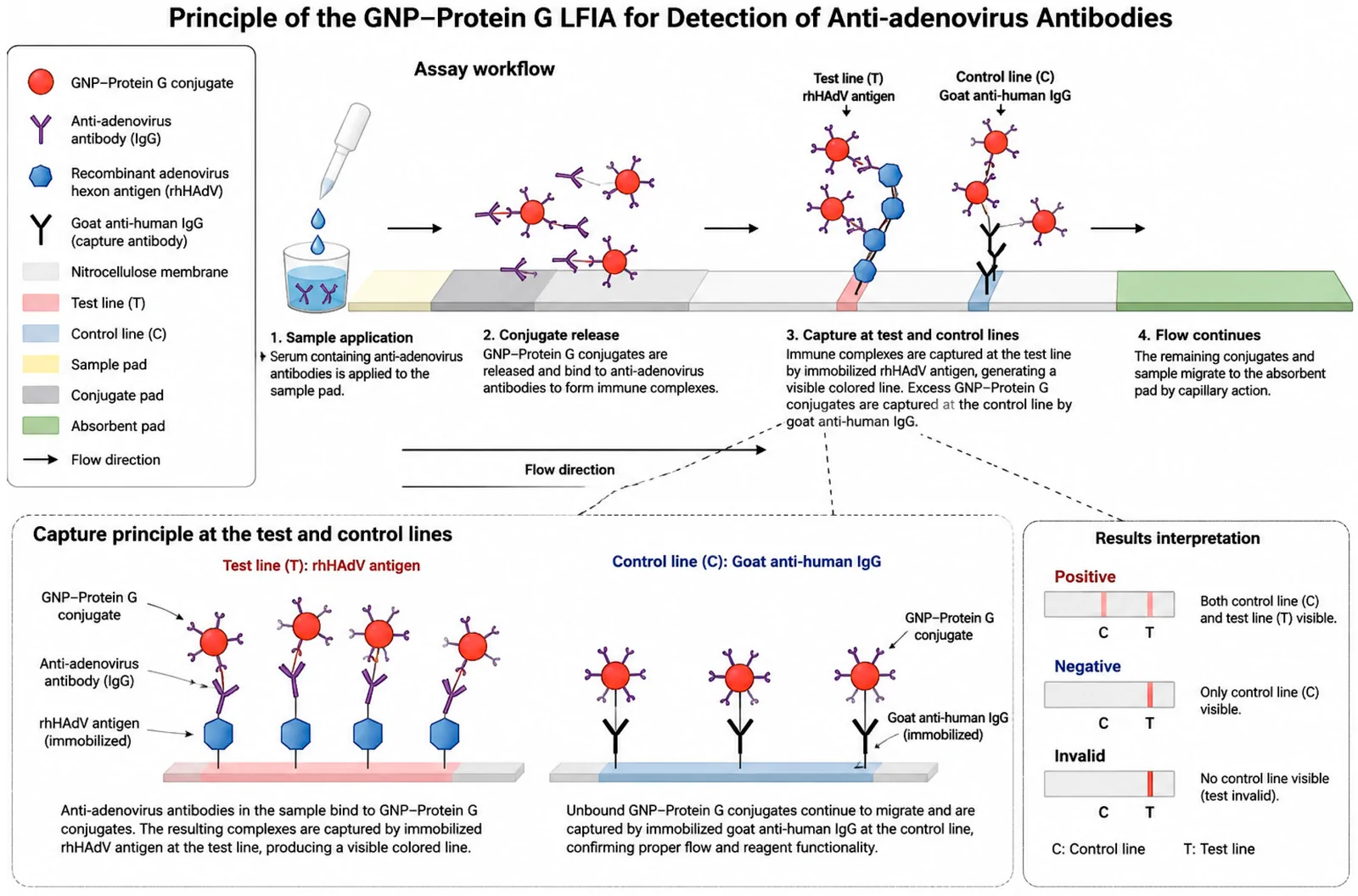
Schematic illustration of the principle of the GNP–Protein G-based lateral flow immunochromatographic assay (LFIA) for the detection of anti-human adenovirus antibodies. Anti-adenovirus antibodies present in the serum bind to the GNP–Protein G conjugate and migrate along the nitrocellulose membrane by capillary action. Immune complexes are captured by immobilized recombinant human adenovirus hexon protein (rhHAdV) at the test line (T), whereas excess GNP–Protein G conjugate is captured at the control line (C), confirming proper assay performance.

The membranes with deposited reagents were dried at 23–25 °C for 20 h in a vacuum drying chamber. After drying, the membrane components were assembled into immunochromatographic composites and cut into 4 mm wide strips using a CM4000 guillotine cutter module (BioDot). The membrane components were assembled at 23–25 °C in a controlled environment with a relative humidity of 25–30%. The principle of the GNP–Protein G-based LFIA is illustrated in Figure 1.

### 2.7. Detection of Antibodies Against rhHAdV

The LFIA was performed at 25 °C. Briefly, 20 µL of the serum sample was mixed with 80 µL of buffer (Tris, pH 7.5; 150 mM NaCl; 0.1% Tween 20; 0.5% BSA) in an Eppendorf tube. A test strip was then vertically immersed into the mixture. The results were evaluated after 20 min. For the GNP-based LFIA, results were interpreted visually under ambient laboratory lighting after 20 min. For the QD-based LFIA, fluorescence was evaluated qualitatively after 20 min using a handheld ultraviolet lamp with an excitation wavelength of 302 nm. The presence or absence of fluorescence at the test line was determined by visual inspection. No dedicated fluorescence reader, emission filter, image acquisition system, or quantitative fluorescence analysis was used. All results were interpreted qualitatively as positive or negative based on the visible presence of the fluorescent test line. Serum samples were classified as positive or negative according to the manufacturer’s instructions for the commercial anti-human adenovirus IgG ELISA, which served as the reference method. The LFIA was interpreted qualitatively. The presence of both the test line and the control line was considered a positive result, whereas the presence of the control line alone indicated a negative result. No quantitative cut-off value or image-based intensity threshold was established.

### 2.8. Selection of Clinical Serum

Archived human serum specimens stored in the laboratory collection of the National Center for Biotechnology were used in this study. The samples had originally been collected for routine diagnostic purposes and were subsequently preserved as part of the institutional serum collection. Before inclusion in the present study, all specimens were irreversibly de-identified, and each serum sample represented a single individual.

All available serum samples that tested positive for anti-adenovirus IgG by a commercial ELISA (*n* = 33) were included in the evaluation panel. In addition, 57 ELISA-negative serum samples were randomly selected from the same archived collection. Sample selection was completed before LFIA testing, and the resulting case–control panel (33 ELISA-positive and 57 ELISA-negative samples) was used to evaluate the agreement of the developed LFIA systems with the reference ELISA.

Because archived irreversibly de-identified serum samples were used, detailed demographic and clinical information, including age, sex, clinical diagnosis, immune status, vaccination history, previous adenovirus infection, indication for testing, sampling date, and geographic origin, was unavailable. Therefore, the study was designed to evaluate the analytical performance and agreement of the developed LFIA systems with the reference ELISA rather than their performance in specific clinical populations.

### 2.9. Statistical Analysis

Agreement between the developed LFIA systems and the reference ELISA was evaluated by calculating positive percent agreement (PPA), negative percent agreement (NPA), Cohen’s kappa coefficient, and corresponding 95% confidence intervals. The 95% confidence intervals for PPA and NPA were calculated using the Wilson score method. Cohen’s kappa coefficient and its 95% confidence interval were calculated from the complete 2 × 2 contingency tables using the GraphPad QuickCalcs “Quantify agreement with kappa” online calculator (version 9.0, GraphPad Software, San Diego, CA, USA).

## 3. Results

### 3.1. Expression and Purification of rhHAdV

**Figure 2:**
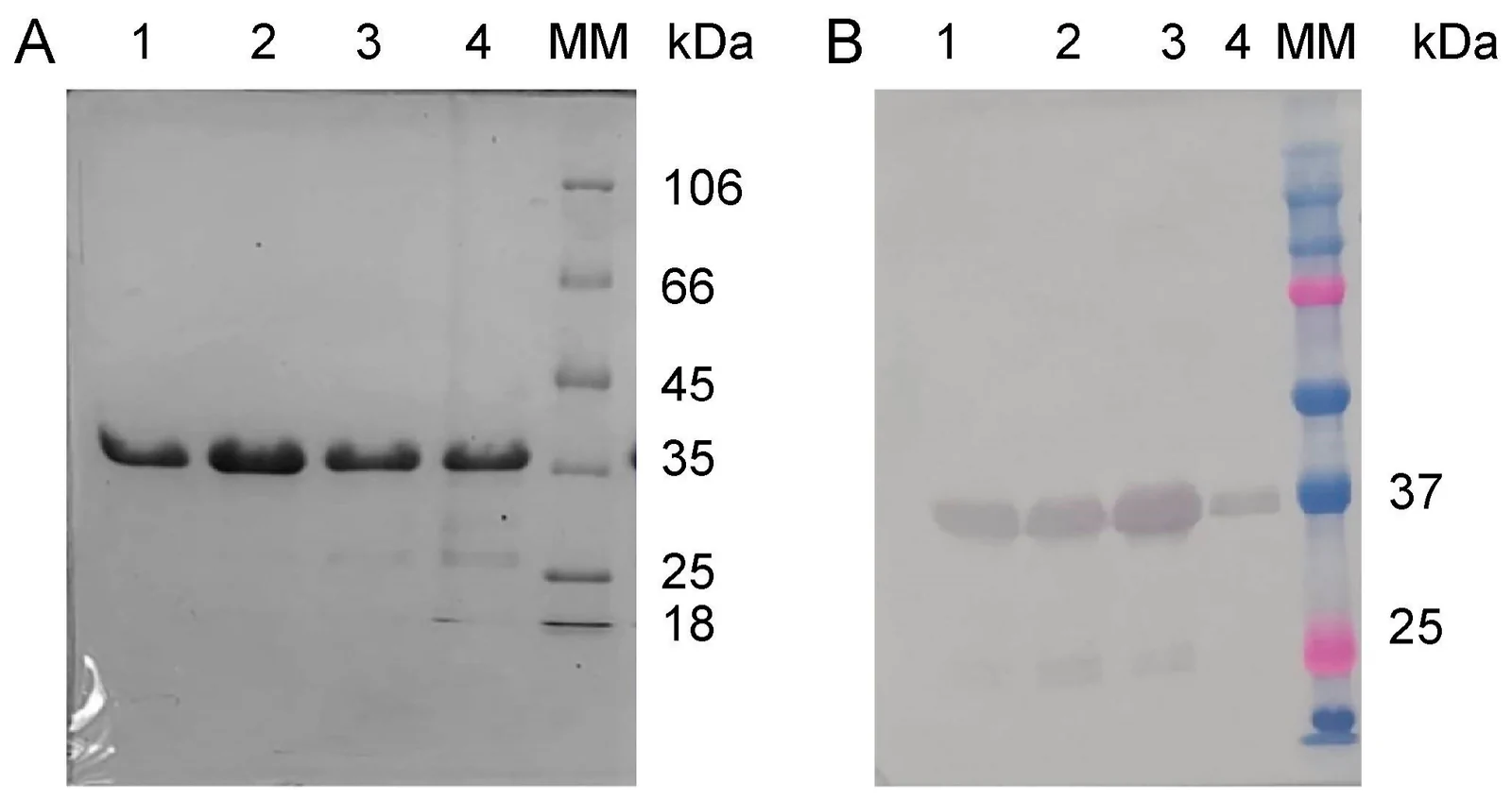
Sodium dodecyl sulfate–polyacrylamide gel electrophoresis (SDS-PAGE) (**A**) and Western blot (**B**) analyses of recombinant human adenovirus hexon protein fragment (rhHAdV) following chromatographic purification. Lanes 1–4, protein purification fractions; MM, molecular weight marker.

Transformation of the pET28/hHAdV plasmid resulted in the generation of an *E. coli* BL21/pET28/hHAdV strain producing a recombinant fragment of the HAdV hexon protein spanning amino acid residues A120 to R316. The expression analyses demonstrated the production of a protein with an approximate molecular weight of 35 kDa. Optimization of expression and purification parameters enabled the isolation of purified rhHAdV protein. The recombinant antigen was purified using nickel-based metal–chelate affinity chromatography. Elution was performed using buffers containing varying concentrations of imidazole. SDS-PAGE revealed a single protein band in the purified preparation, indicating a high level of purity (Figure 2).

As shown in Figure 2A, the optimized expression and purification protocol enabled the production of rhHAdV preparations that appeared predominantly homogeneous on Coomassie-stained SDS-PAGE. Western blot analysis using an anti-His-tag monoclonal antibody detected a single immunoreactive band with an apparent molecular weight of approximately 35 kDa, consistent with the expected size of the His-tagged recombinant HAdV-B3 hexon fragment (Figure 2B).

**Figure 3:**
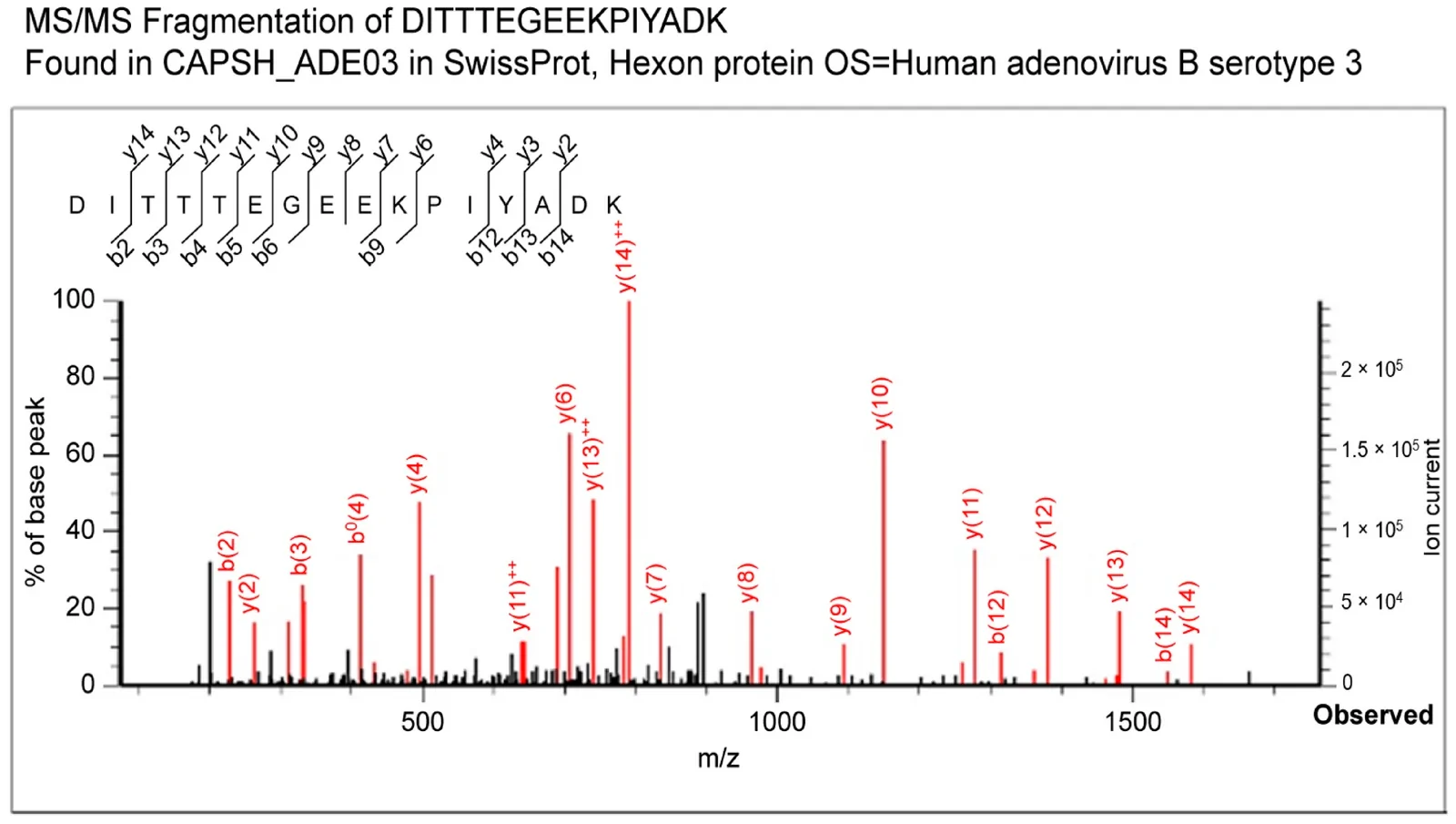
Liquid chromatography–tandem mass spectrometry (LC-MS/MS) analysis of the recombinant human adenovirus hexon protein fragment (rhHAdV).

The identity of the recombinant protein was further supported by LC-MS/MS analysis. Mascot database searching identified the peptide DITTTEGEEKPIYADK corresponding to the rhHAdV, with a maximum Mascot score of 1206 (Figure 3). Together with the expected molecular weight and anti-His immunoblotting results, these findings support the identity of the expressed recombinant protein. Detailed proteomic characterization, including sequence coverage, post-translational modification analysis and intact mass determination, was beyond the scope of the present study.

### 3.2. Immunoreactivity of rhHAdV in ELISA with Human Positive Sera

**Figure 4:**
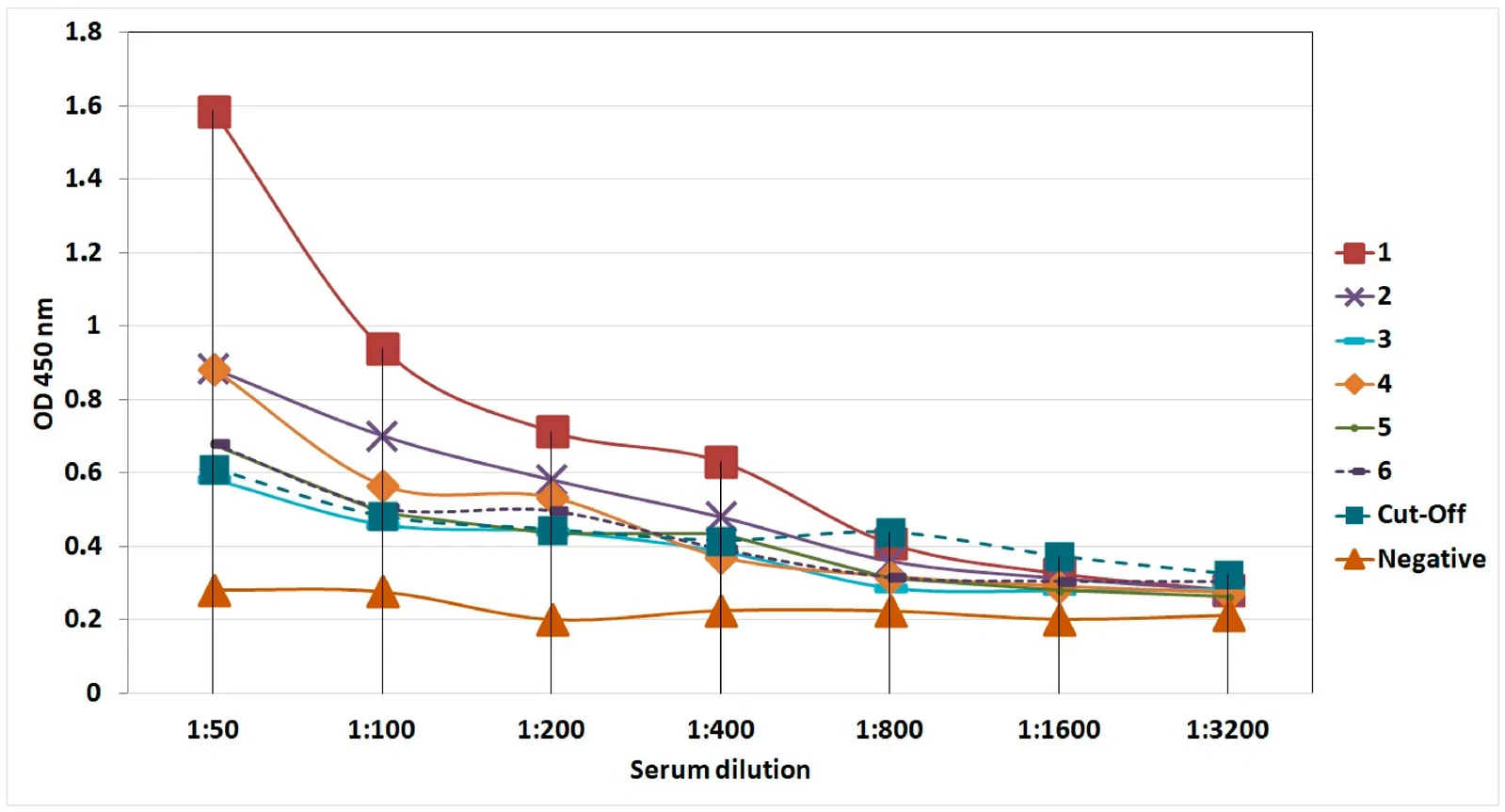
ELISA reactivity of six representative human serum samples against the recombinant human adenovirus hexon protein fragment (rhHAdV) at serial serum dilutions (1:50–1:3200). Curves 1–6 represent six individual positive human serum samples. The cut-off line indicates the assay positivity threshold, whereas the negative curve represents the negative control serum. The y-axis shows the optical density (OD450) measured in the ELISA.

The immunoreactivity of rhHAdV was evaluated by ELISA using seven representative serum samples selected from the final evaluation panel. Six samples were ELISA-positive, and one was ELISA-negative. Representative ELISA reactivity is shown in Figure 4.

All available ELISA-positive serum samples (*n* = 33) were included in the comparative evaluation panel. This panel included ELISA-positive sera with relatively low antibody reactivity, allowing assessment of LFIA performance in samples with lower antibody levels. The same serum panel was subsequently used for comparative evaluation of the GNP- and QD-based LFIA formats. The representative serum samples shown in Figure 4 were part of this final evaluation panel.

### 3.3. Optimization of LFIA Components

**Figure 5:**
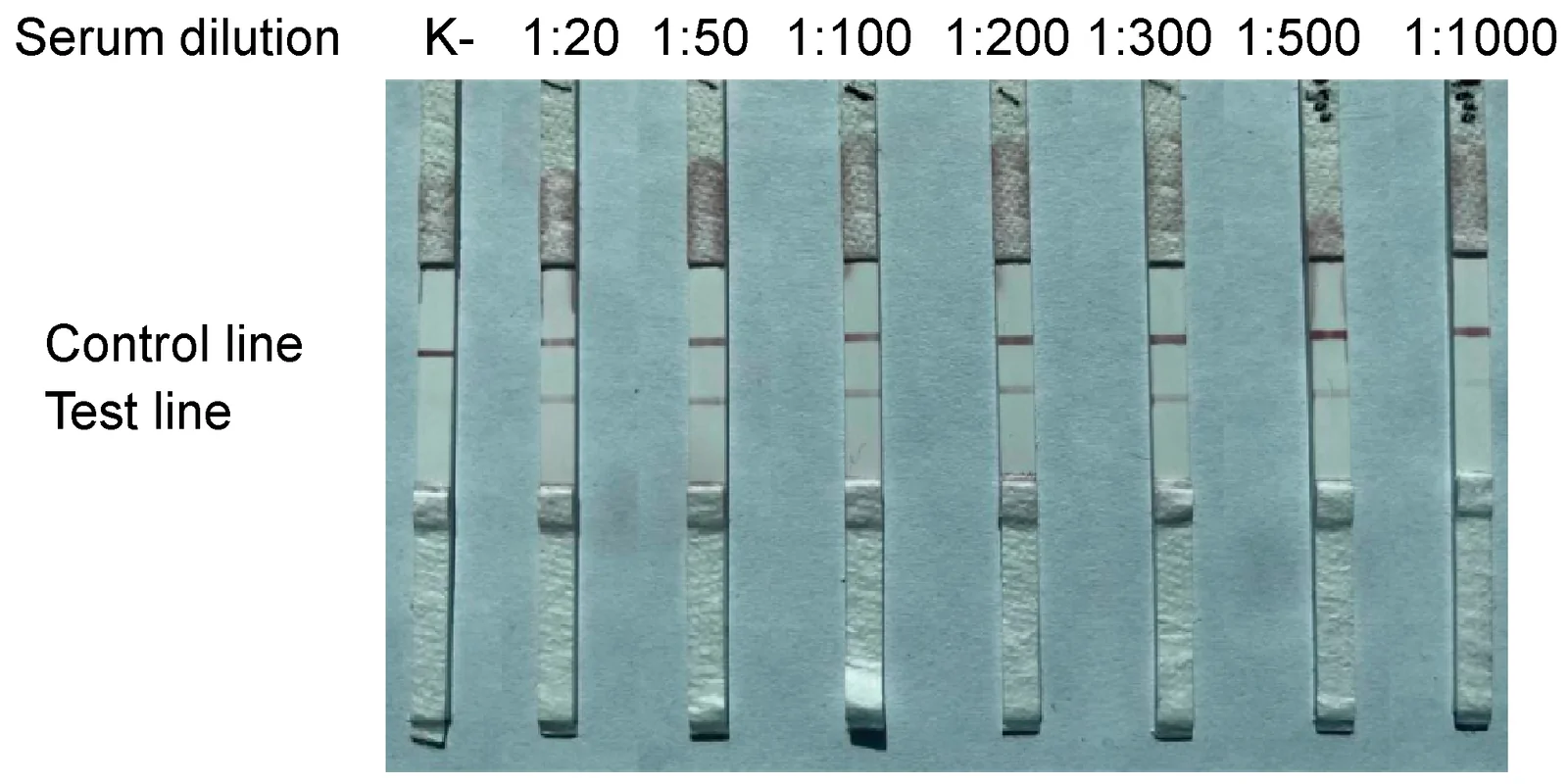
Lateral flow immunochromatographic assay (LFIA) of serially diluted serum samples using the gold nanoparticle–Protein G conjugate (GNP-G) for evaluation of antibody detection at serial serum dilutions.

GNP-G was prepared following the methods described by Sotnikov et al. [13]. The LFIA was assembled using a CNPC-SS12-L2-H50 nitrocellulose membrane and PT-R5 conjugate pad. A working concentration of 1 mg/mL rhHAdV was used for immobilization on the test line in all subsequent experiments. The GNP-G conjugate was applied to the PT-R5 membrane at a volume of 11 µL/cm. Control sera were tested at a dilution of 1:20. The dilutional detectability of the optimized assay was evaluated using serial dilutions of a single positive serum sample (1:20–1:1000). The highest serum dilution yielding a clearly visible positive test line was 1:300 for the GNP-based LFIA (Figure 5).

**Figure 6:**
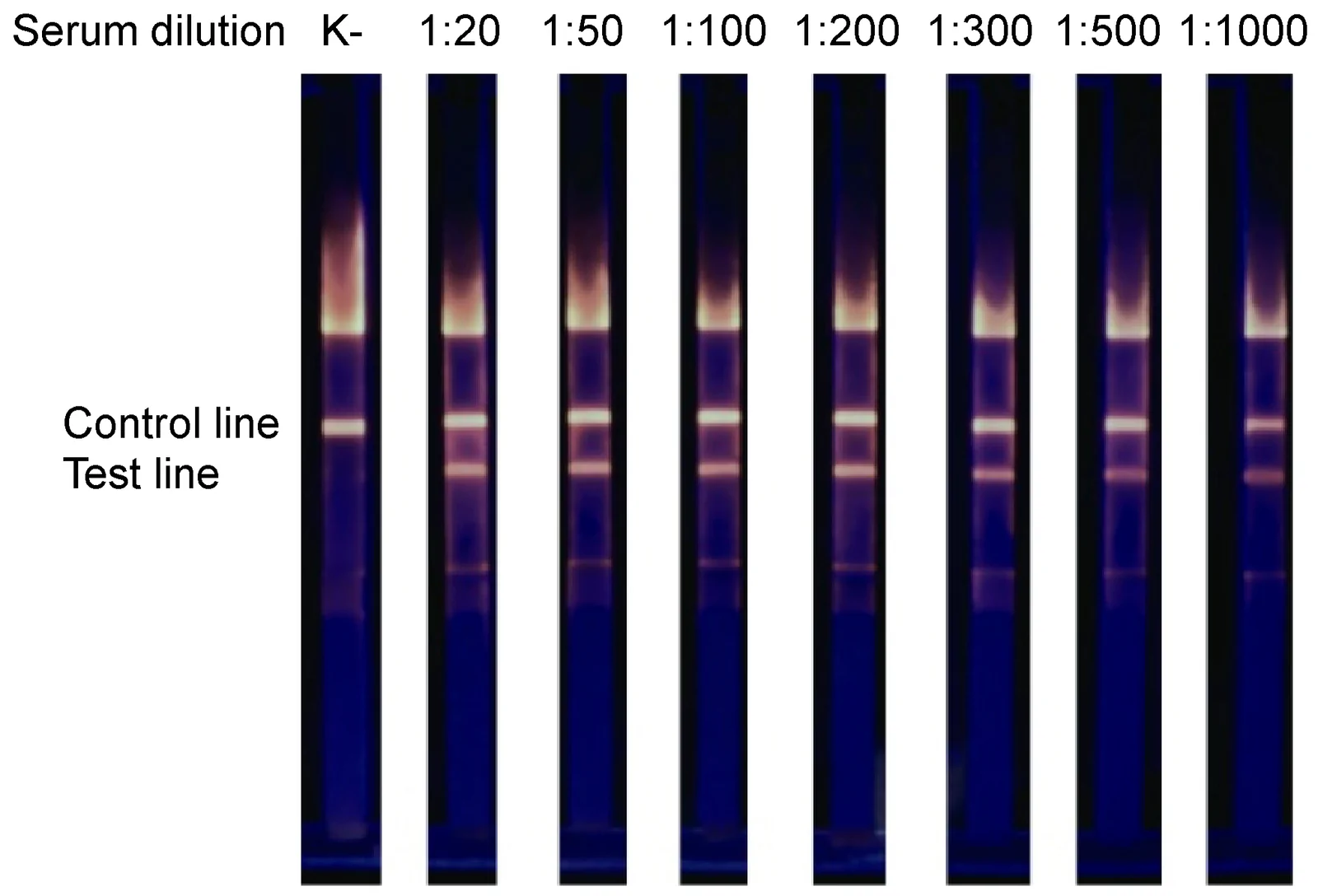
Lateral flow immunochromatographic assay (LFIA) using the quantum dot–Protein G conjugate (QD-G) for evaluation of antibody detection at serial serum dilutions.

The analytical performance of the LFIA based on QD-G was evaluated using the same concentrations of rhHAdV and identical serum dilutions as those applied for the GNP-based assay. For the QD-based LFIA, the test signal remained visually detectable up to a serum dilution of 1:1000 (Figure 6).

For subsequent experiments, a serum dilution of 1:20 was selected, as it ensured clear and intense visualization of both test and control lines when analyzing positive samples, while only the control line was observed for negative sera. These findings demonstrated visually detectable antibody signals over the tested serum dilution range, with a higher endpoint dilution observed for the QD-based LFIA.

### 3.4. Agreement of GNP- and QD-Based LFIAs with the Reference ELISA

The performance of the LFIAs based on GNP and QD conjugates was evaluated using a panel of 90 serum samples. A commercial ELISA kit for the detection of human adenovirus IgG antibodies was used as the reference. Among 57 ELISA-negative serum samples, both LFIAs yielded negative results in 56 cases, corresponding to an NPA of 98.2% (Table 1).

Among the 33 positive samples, antibodies were detected in 32 samples using the GNP-G conjugate and in all 33 samples using the QD-G conjugate. The numbers of correctly classified positive and negative samples are presented in Table 2. The diagnostic performance of both LFIA systems, including positive percent agreement, negative percent agreement, Cohen’s kappa coefficient, and 95% confidence intervals, is summarized in Table 3. The PPA values for the GNP-G and QD-G LFIAs were 96.9% and 100%, respectively. Both LFIA formats showed high agreement with the reference ELISA. Although the QD-based assay detected one additional positive sample compared with the GNP-based assay, the study was not powered to determine whether this difference was statistically significant.

Both assays showed an NPA of 98.2%, while the QD-G assay showed a numerically higher PPA than the GNP-G assay (100% vs. 96.9%). Cohen’s kappa analysis demonstrated almost perfect agreement between both LFIA formats and the reference ELISA. The GNP-G assay showed a κ value of 0.952, whereas the QD-G assay demonstrated a κ value of 0.976.

## 4. Discussion

In the present study, the rhHAdV was successfully expressed, purified, and evaluated as an antigen for serological detection of anti-adenovirus antibodies. Both GNP- and QD-based LFIA formats demonstrated high agreement with the reference ELISA, whereas the QD-based assay showed greater dilutional detectability in the serum dilution experiment. These findings support the feasibility of fluorescence-assisted LFIA for rapid serological assessment of previous adenovirus exposure. Rapid serological methods are valuable for epidemiological surveillance and seroprevalence studies. Compared with conventional laboratory assays such as ELISA, LFIA provides a rapid and simple alternative requiring minimal sample preparation and laboratory infrastructure [14,15]. The recombinant HAdV-B3 hexon fragment (A120-R316) was selected based on published information describing the antigenic organization of the adenovirus hexon protein and its suitability for recombinant expression [16,17]. Although this region has been reported to contain immunoreactive domains, structural modeling, sequence conservation analysis, and experimental B-cell epitope mapping were beyond the scope of the present study. Previous studies have demonstrated the suitability of recombinant viral antigens for serological applications [18,19]. Consistent with these findings, the rhHAdV demonstrated immunoreactivity in both ELISA and LFIA using positive human sera. The rhHAdV was expressed in *E. coli* and was used as a recombinant serological antigen. Its three-dimensional conformation was not evaluated in the present study and may differ from that of the corresponding region in the native viral capsid. No SEC, DLS or other biophysical characterization of refolding efficiency was performed. Nevertheless, its recognition by anti-adenovirus antibodies in Western blotting, ELISA, and LFIA indicates that the recombinant fragment retained antigenic determinants suitable for antibody detection.

Both LFIA formats showed high agreement with the reference ELISA. Although the QD-based assay detected one additional positive sample compared with the GNP-based assay, this difference was based on a single specimen and should therefore be interpreted cautiously. The limited sample size does not allow conclusions regarding superior clinical performance of the QD-based assay. However, in the serum dilution experiment, the QD-based LFIA retained a visually detectable signal at a higher serum dilution (1:1000) than the GNP-based format (1:300), indicating greater dilutional detectability under the conditions tested. This observation is consistent with previous reports describing the advantages of quantum dots as fluorescent labels because of their high fluorescence intensity and photostability [9,20]. Unlike the GNP-based assay, which could be interpreted under ambient laboratory lighting, the QD-based LFIA required visualization using a handheld 302 nm ultraviolet lamp. Although no dedicated fluorescence reader was required, the QD-based assay cannot be considered completely instrument-free.

Because the commercial anti-human adenovirus IgG ELISA does not disclose the exact antigen composition or adenovirus serotypes represented in its coating antigen, agreement between the ELISA and the rhHAdV-based LFIA should not be interpreted as evidence of HAdV-B3-specific antibody detection. Rather, the observed agreement reflects concordance between two serological assays under the conditions of this study. Confirmation of HAdV-B3-specific diagnostic performance will require validation using well-characterized HAdV-B3-positive sera and, where appropriate, virus neutralization assays.

The commercially available Qdot™ 585 nanocrystals used in this study may contain cadmium-based semiconductor materials. However, environmental safety, disposal requirements, and biosafety considerations associated with their use were beyond the scope of the present proof-of-concept study and warrant dedicated investigation before potential point-of-care implementation.

The present assay was designed to detect binding IgG antibodies against the rhHAdV and is therefore intended primarily for the serological assessment of previous adenovirus exposure. It is not intended for the diagnosis of acute adenovirus infection, determination of infectiousness, or assessment of neutralizing immunity against adenoviral vectors. Because the assay detects binding rather than neutralizing antibodies, additional studies correlating LFIA results with virus neutralization assays will be required before its potential application in evaluating protective immunity or pre-existing immunity to adenoviral vectors can be considered. Protein G binds predominantly to the Fc region of human IgG subclasses, particularly IgG1, IgG2, and IgG4. In the present study, assay performance was evaluated exclusively using serum samples classified according to a commercial anti-human adenovirus IgG ELISA. Therefore, within the scope of this study, the LFIA results were interpreted as reflecting anti-adenovirus IgG antibody detection. Although Protein G exhibits preferential binding to human IgG, the isotype specificity of the developed LFIA was not experimentally evaluated in the present study. Therefore, the assay should be interpreted as a serological method evaluated against a commercial anti-human adenovirus IgG ELISA rather than as an experimentally validated IgG-specific assay. Further studies using purified immunoglobulin isotypes will be required to confirm isotype specificity.

Several limitations should be acknowledged. The study was performed using a relatively small retrospectively assembled case–control serum panel lacking detailed demographic and clinical information. In addition, LFIA results were interpreted with knowledge of the reference ELISA classification; therefore, observer bias cannot be completely excluded. The rhHAdV was selected based on published information rather than structural modeling or experimental epitope mapping, and its conformational integrity after purification under denaturing conditions was not evaluated. Dilutional detectability was assessed using serial dilutions of a single positive serum sample and should not be interpreted as a formal analytical limit of detection. Physicochemical characterization of the GNP–Protein G and QD–Protein G conjugates, including particle size, polydispersity, zeta potential, conjugation efficiency, fluorescence retention, colloidal stability, and batch-to-batch variability, was beyond the scope of this proof-of-concept study. Consequently, the observed differences between the GNP- and QD-based LFIA formats should be interpreted as comparative assay performance rather than as a mechanistic evaluation of nanoparticle properties. In addition, the proof-of-concept nature of the study did not include formal analytical validation, including repeatability, reproducibility, robustness, shelf-life stability, evaluation of different biological sample matrices, analytical specificity, cross-reactivity with other adenovirus serotypes or viral infections, or potential interference from endogenous factors. Therefore, the reported diagnostic specificity reflects agreement with the reference ELISA rather than comprehensive analytical specificity. Future studies should include larger prospective multicenter cohorts, structural characterization of the recombinant antigen, physicochemical characterization of nanoparticle conjugates, comprehensive analytical validation, and systematic cross-reactivity and interference studies.

Overall, the rhHAdV proved to be a suitable antigen for serological detection of anti-adenovirus antibodies by LFIA. The QD-based format demonstrated greater dilutional detectability while maintaining high agreement with the reference ELISA. The results indicate the feasibility of fluorescence-assisted LFIA for serological and epidemiological applications. However, further analytical and clinical validation will be required before routine diagnostic implementation or commercialization can be recommended.

## 5. Conclusions

The developed LFIA provides a rapid method for detecting binding antibodies reactive with the recombinant HAdV-B3 hexon fragment and may be useful for serological studies. Further studies are required to determine its relationship with neutralizing antibody responses. Both the GNP- and QD-based LFIA formats demonstrated high agreement with the reference ELISA. The QD-based LFIA showed a higher endpoint dilution in the tested serum while maintaining comparable agreement with the reference ELISA. These findings demonstrate the feasibility of using the rhHAdV in combination with QD-based detection for qualitative serological assessment of anti-adenovirus antibodies. Further analytical and clinical validation is required before broader application can be considered.

## Figures and Tables

**Table 1:** Agreement of GNP-G and QD-G lateral flow immunochromatographic assays with the reference ELISA.

Parameter	Correct/Total	Value (%)	95% CI
Positive Percent Agreement (PPA)(GNP-G)	32/33	96.9	84.7–99.5
Positive Percent Agreement (PPA) (QD-G)	33/33	100	89.6–100
Negative Percent Agreement (NPA) (GNP-G and QD-G)	56/57	98.2	90.7–99.7

CI, confidence interval; GNP-G, gold nanoparticle–Protein G conjugate; QD-G, quantum dot–Protein G conjugate.

**Table 2:** Comparison of LFIA results with the reference ELISA.

ELISA Result	GNP-G Negative	GNP-G Positive	QD-G Negative	QD-G Positive
Negative (*n* = 57)	56	1	56	1
Positive (*n* = 33)	1	32	0	33

ELISA, enzyme-linked immunosorbent assay; LFIA, lateral flow immunochromatographic assay; GNP-G, gold nanoparticles + Protein G conjugate; QD-G, quantum dot + Protein G conjugate.

**Table 3:** Agreement of the developed LFIA systems with the reference ELISA.

Parameter	GNP-G	QD-G
Positive Percent Agreement (%)	96.9	100
Negative Percent Agreement (%)	98.2	98.2
Cohen’s κ	0.952	0.976
95% CI	0.887–1.000	0.930–1.000

## Data Availability

The data presented in this study are available from the corresponding author upon reasonable request.

## Supplementary Materials

The following supporting information can be downloaded at https://www.mdpi.com/article/10.3390/diagnostics16162670/s1, Table S1: Amino acid sequence of the rHAdV-B3 protein fragment (A120-R316) used in this study. Table S2: The complete codon-optimized nucleotide sequence of the synthetic gene.

## Abbreviations

The following abbreviations are used in this manuscript:

BSA	Bovine serum albumin
EDC	*N*-(3-dimethylaminopropyl)-*N*′-ethylcarbodiimide hydrochloride
ELISA	Enzyme-linked immunosorbent assay
GNP	Gold nanoparticle
GNP-G	Gold nanoparticle–Protein G conjugate
HAdV	Human adenovirus
H-FABP	Human fatty acid-binding protein
IPTG	Isopropyl-β-d-1-thiogalactopyranoside
LFIA	Lateral flow immunochromatographic assay
MES	2-(*N*-morpholino)ethanesulfonic acid buffer
NHS	*N*-hydroxysulfosuccinimide sodium salt
PBS	Phosphate-buffered saline
PCR	Polymerase chain reaction
rhHAdV	Human adenovirus hexon protein fragment
QD	Quantum dot
QD-G	Quantum dot–Protein G
SDS-PAGE	Sodium dodecyl sulfate–polyacrylamide gel electrophoresis
