# Supplementary material for: Lateral Flow Immunochromatographic Assay for Detection of Antibodies Against a Recombinant Human Adenovirus Type 3 Hexon Fragment Using Gold Nanoparticles and Quantum Dots
Source: Diagnostics (Basel). 2026 Aug 21;16(16):2670. doi: 10.3390/diagnostics16162670 (PMC13512234; doi:10.3390/diagnostics16162670)
Supplement: Supplementary file 1 [file diagnostics-16-02670-s001.zip › diagnostics-4451654-supplementary.pdf]

diagnostics

MDPI

Supplementary Materials:

Supplementary Table S1. Amino acid sequence of the rHAdV-B3 protein fragment (A120–R316) used in this study.

Name	GenBank accession No.	Protein sequence
Hexon, partial [Human adenovirus B3]	BAF42828.1	aynslapkga pntsqwivtt nrdnavtttt ntfgiasmkg dnitkeglqi gkditttege ekpiyadkty qpepqvgees wtdtdvtnek fggralkpat nmkpcygsfa rptnikggqa knrkvkptte ggveieepdi dmeffdgrda vagalapeiv lytenvnlet pdshvvykpg tsdnshanlg qqampn

Supplementary Table S2. The complete codon-optimized nucleotide sequence of the synthetic gene.

Plasmide	codon-optimized nucleotide sequence
pET-28a(+)	atgggcagcagccatcatcatcatcatcacagcagcggcctggtgccgcgcggcagccatatggctagcatgactggtggacagcaaatgggtc ggatccgaattcccatgggtgcgtataacagcctggcgccgaaaggcgcgccgaacaccagccagtggattgtgaccaccaaccgcgataacgc ggtgaccaccaccaccaacacctttggcattgcgagcatgaaaggcgataacattaccaaagaaggcctgcagattggcaaagatattaccaccac cgaaggcgaagaaaaaccgatttatgcggataaaacctatcagccggaaccgcaggtgggcgaagaaagctggaccgataccgatgtgacca acgaaaaatttggcggccgcgcgctgaaaccggcgaccaacatgaaaccgtgctatggcagctttgcgcgcccgaccaacattaaaggcggcca ggcgaaaaaccgcaaagtgaaaccgaccaccgaaggcggcgtggaaattgaagaaccggatattgatatggaatttttgatggccgcgatgcg gtggcgggcgcgctggcgccggaaattgtgctgtataccgaaaacgtgaacctggaaaccccggatagccatgtggtgtataaaccgggcacca gcgataacagccatgcgaacctgggccagcaggcgatgccgaatctcgagcaccaccaccaccaccac

 https://doi.org/10.3390/diagnostics16162670
